# Stimuli changes and challenging behavior in nursing homes during the COVID-19 pandemic

**DOI:** 10.1186/s12877-022-02824-y

**Published:** 2022-02-19

**Authors:** Inge A. H. Knippenberg, Ruslan Leontjevas, Johanna M. H. Nijsten, Christian Bakker, Raymond T. C. M. Koopmans, Debby L. Gerritsen

**Affiliations:** 1 Department of Primary and Community Care, Radboud University Medical Center, Radboud Institute for Health Sciences, Radboudumc Alzheimer Center, Nijmegen, The Netherlands; 2 Faculty of Psychology, Open University of the Netherlands, Heerlen, The Netherlands; 3 Archipel, Center for Specialized Geriatric Care, Eindhoven, The Netherlands; 4 Groenhuysen, Center for Specialized Geriatric Care, Roosendaal, The Netherlands; 5 Joachim en Anna, Center for Specialized Geriatric Care, Nijmegen, The Netherlands

**Keywords:** Nursing home, COVID-19, Challenging behavior, Stimuli, Activities

## Abstract

**Background:**

COVID-19 restrictions in nursing homes resulted in a reduction in stimuli for residents. This study aimed to explore observed effects of changes in stimuli, both targeted (e.g., planned recreational activities) and untargeted (e.g., spontaneous noise), on challenging behavior in nursing home residents during COVID-19 anti-pandemic measures.

**Methods:**

In an online survey, nursing home healthcare professionals in the Netherlands provided their perspectives on the effects of the reduction in untargeted stimuli on residents with mild, advanced, or no dementia, and on different types of challenging behavior (i.e., psychotic, depressed, anxious, agitated, or apathetic behavior). Additionally, we asked participants’ opinions about strategies for limiting untargeted stimuli and for adjusting targeted stimuli for optimal management of challenging behaviors.

**Results:**

In total, 199 professionals completed the survey. Residents with advanced dementia and those with psychotic and agitated behavior seemed to benefit from the reductions in stimuli not specifically targeted at the resident. In contrast, residents without dementia and those with depressive and apathetic behavior seemed to be negatively affected by reductions in untargeted stimuli. Participants would like to continue reducing untargeted stimuli in the future (e.g., limiting the use of corridors adjacent to residents' rooms) and to adapt existing or introduce new initiatives involving targeted stimuli (e.g., small-scale, individually tailored activities). Responses to open-ended questions revealed additional initiatives that could be useful in nursing home care.

**Conclusions:**

This study provided lessons to learn from the COVID-19 measures in nursing homes. While many residents may have been negatively affected by the restrictions imposed during the pandemic, specific resident groups may have benefitted from the reduction in untargeted stimuli and from the adjustments made to daily activities. Various strategies and initiatives used in nursing homes during the pandemic seem promising for meeting individual needs in managing challenging behavior. These findings suggest that certain stimuli may affect specific resident groups differently. This underlines the importance of finding the right balance between stimuli and tranquility, tailored to the needs of individual residents. It is important to consider the stimuli present in nursing homes, whether targeted or untargeted, when analyzing and treating challenging behavior.

## Background

Challenging behavior is common in nursing home (NH) residents, especially in those with dementia [1]. Previous studies suggest that environmental stimuli can influence challenging behavior in residents [2–4]. In our prior study, NH staff consistently reported that changes in such stimuli due to COVID-19 restrictions affected challenging behaviors in different ways [5]. The experiences and insights of healthcare professionals who observed the effects of the COVID-19 restrictions on challenging behavior in specific resident groups can be used to improve future NH care.

To limit the spread of COVID-19, the Dutch government imposed a nationwide ban on NH visits in the Netherlands, which took effect on March 19, 2020 [6]. In addition, most non-care-related activities, such as recreational activities, were discontinued or adjusted in many NHs. It has been recognized that COVID-19 measures have resulted in negative psychological and behavioral consequences for older adults with and without dementia [7–14]. Likewise, studies in long-term care settings have found increases in depression, anxiety, loneliness, and behavioral problems that were attributed to the COVID-19 restrictions [15–21].

On the other hand, some studies have suggested that pandemic measures may have had neutral or positive effects and may have been associated with no changes in behavior or even with improved behavioral outcomes for some residents. For example, one study found that the majority of people with Alzheimer's disease showed no changes in neuropsychiatric symptoms during the quarantine [22]. Moreover, our study suggested both increased and decreased challenging behavior in NH residents while the COVID-19 restrictions were in place [5].

A possible explanation for these results is that particular resident groups (e.g., residents with or without dementia) experienced the effects of the COVID-19 restrictions differently [5, 16, 17]. As residents with dementia are more susceptible to sensory overstimulation [2, 3, 23], it can be argued that the reductions in unintentional stimuli (e.g., loud noises in the corridors) as a result of the restrictions may have had a beneficial effect on some residents. Additionally, to mitigate the effects of these restrictions, many NHs deployed adjusted or new initiatives, such as providing individual activities instead of group activities and providing support for online communication between residents and their relatives [5, 24–27]. Some of these strategies may have had beneficial effects on challenging behavior and may possibly continue to do so, even in a post COVID-19 era.

The measures taken to slow the spread of the COVID-19 virus provided a unique situation. The experiences of NH professionals may reveal important lessons for future management of challenging behavior in particular resident groups. Hence, this study aimed to explore NH professionals’ opinions and observations of the way changes in stimuli brought about by the COVID-19 restrictions affected specific groups (i.e., residents with no, mild, or advanced dementia) and different types of challenging behavior (i.e., psychotic, depressed, anxious, agitated, or apathetic). This study also analyzed professionals’ perceptions of proposals to limit stimuli in the future, and opinions about whether adjustments made to certain stimuli as a result of the COVID-19 restrictions and the new activities implemented during this time should be kept after the pandemic. In this study, we distinguish between stimuli specifically targeted at residents, such as organized activities, and stimuli not specifically targeted at residents, such as unintentional noise in the corridors.

## Methods

### Study Design and Procedure

An online survey was conducted among NH professionals between November 10, 2020 and January 22, 2021, during the second wave of the pandemic in the Netherlands. We used three methods to recruit participants. First, we invited those NH professionals who had participated in the previous study and had given us permission to contact them for a follow-up survey. Second, we recruited NH activity therapists by contacting a selection of Dutch NH organizations (specifically, every sixth organization in a comprehensive list of Dutch NHs). Finally, we recruited additional participants (psychologists, elderly care physicians, nurse specialists, and activity therapists) through our LinkedIn and professional networks.

### Ethics

This study adhered to the World Medical Association Declaration of Helsinki (2013) [28] as well as relevant applicable guidelines in the Netherlands. All participants were informed about the aim of the study and provided online informed consent. Data remained anonymous during the collection, analysis, and storage processes. According to the guidelines of the Medical Ethics Review Committee at the Radboud university medical center Nijmegen, the Netherlands, the study does not fall under the scope of the Dutch Medical Research Involving Human Subjects Act (WMO) [29].

### Survey

The survey was developed using topics that emerged from a previous study conducted during the pandemic’s first wave about the extent to which anti-pandemic measures affected challenging behavior [5]. In the current study, we asked participants to provide their demographic information such as age and gender, as well as their observations regarding the effects of COVID-19 measures on specific resident groups within three topics: (1) stimuli that were not specifically targeted at the resident (untargeted stimuli), (2) stimuli that were specifically targeted at the resident (targeted stimuli), and (3) online communication. The latter topic was analyzed in a separate study [30].

A structured questionnaire was used to collect participants’ observations of the effects of stimuli changes on challenging behavior in residents and participants’ opinions about whether to retain certain strategies within these topics (e.g., limiting the use of corridors adjacent to residents' rooms by suppliers and staff as strategy to reduce untargeted stimuli). In the instructions of this questionnaire, we provided a definition and examples of untargeted stimuli (i.e., “Events that take place around a resident, without being specifically targeted at the resident. For example, people walking down the corridor or actions that take place in communal areas.”) and targeted stimuli (i.e., “Targeted interaction with the resident refers to events in which the resident is involved purposefully. For example, provided activities, therapies, or visits from loved ones.”). Professionals were given the opportunity, through open-ended questions, to elaborate on their answers, provide in-depth information, or add strategies and ideas to the questionnaire’s predefined categories.

We asked participants to consider three resident groups: those without dementia, those with mild dementia, and those with advanced dementia when responding to questions regarding the effects of the changes in stimuli on residents’ challenging behavior. We also asked participants to consider different types of challenging behavior categorized according to the classifications provided in the Dutch guideline for challenging behavior (i.e., psychotic, depressed, anxious, agitated, or apathetic) [31].

For example, regarding untargeted stimuli, we asked participants to complete a statement on a seven-point scale ranging from “a strong decrease” to “a strong increase”: “Reducing untargeted stimuli leads to [..] in challenging behavior in most residents without dementia.” In terms of targeted stimuli, we asked whether participants wanted to continue the practice of organizing activities in small groups, asking them to respond with one of three options: “yes,” “no,” or “don’t know.”

### Participants

A total of 199 professionals in NH care (78 psychologists, 42 elderly care physicians and nurse specialists, 69 activity therapists, and 10 other professionals) completed the survey. Of those, 181 (91%) were female, and the mean age was 42 years (*SD* = 12.6) (see Table 1).

**Table 1:** Demographic characteristics of participants

	Total (N, 199)	Psychologists (N, 78)	Elderly care physicians and nurse specialists (N, 42)	Activity therapists (N, 69)	Other professionals (N, 10)
Age, mean (SD) [range]	42.3 (12.6) [21–73]	37.0 (10.7) [23–63]	45.0 (12.5) [25–73]	45.8 (12.7) [21–66]	48.3 (12.7) [24–61]
Sex, female, N (%) / male, N	181 (91.0%) / 18	71 (91.0%) / 7	34 (81.0%) / 8	66 (95.7%) / 3	10 (100.0%) / 0
Years working in current organization, median (25–75%) [range]	4 (2–12) [0–40]	4 (2–9) [1–25]	6 (2–12) [0–31]	4 (2–16) [1–40]	7 (2.75–12.75) [1–27]

The other professionals were six nurses, two case managers, a spiritual counselor, and a music therapist

### Analyses

We analyzed participants’ responses to the closed questions through descriptive statistics using SPSS 25. Responses to the open-ended questions were coded using a conventional approach [32] with the help of ATLAS.ti (version 8). One researcher (IK) coded all responses. These codings were checked by another researcher (RL), and, subsequently, interpretations were discussed among the two researchers to reach consensus.

## Results

### Untargeted Stimuli

#### Resident Groups and Type of Challenging Behavior

Two-thirds of the participants (66%) observed an increase in challenging behavior among most residents without dementia when untargeted stimuli decreased because of the COVID-19 restrictions (see Table 2). An even larger number of participants (77%) observed a decrease in challenging behavior under the same circumstances among most residents with advanced dementia. In particular, a decrease in psychotic behavior (51%) and agitated behavior (64%) was observed, while residents’ depressive behavior (73%) and apathetic behavior (69%) increased, according to most participants. Many participants (44%) noticed an increase in residents’ anxious behavior, while others (36%) perceived a decrease.

**Table 2:** Observed changes in challenging behavior in most residents due to a reduction of untargeted stimuli

	N	Decrease	No change	Increase	Don’t know
Challenging behavior per resident group					
No dementia	197	28 (14.2%)	23 (11.7%)	129 (65.5%)	17 (8.6%)
Mild dementia	194	90 (46.4%)	12 (6.2%)	84 (43.3%)	8 (4.1%)
Advanced dementia	195	150 (76.9%)	14 (7.2%)	23 (11.8%)	8 (4.1%)
Type of challenging behavior					
Psychotic	194	99 (51.0%)	37 (19.1%)	35 (18.0%)	23 (11.9%)
Depressive	189	21 (11.1%)	25 (13.2%)	137 (72.5%)	6 (3.2%)
Anxious	194	86 (44.3%)	30 (15.5%)	69 (35.6%)	9 (4.6%)
Agitated	196	126 (64.3%)	10 (5.1%)	55 (28.1%)	5 (2.6%)
Apathetic	198	19 (9.6%)	33 (16.7%)	137 (69.2%)	9 (4.5%)

N participants (%). Participants answered questions regarding observed changes in challenging behavior using a 7-point scale plus the option “don’t know.” The items on the 7-point scale were as follows: 1 = strong decrease, 2 = decrease, 3 = some decrease, 4 = no change, 5 = some increase, 6 = increase, and 7 = strong increase. This table presents percentages for decrease (options 1 to 3), no change (option 4), and increase (options 5 to 7). Participants were not obligated to answer these questions

Most participants reported that changes in the different types of challenging behavior varied between residents without dementia, those with mild dementia, and those with advanced dementia (66% answered “yes,” whereas 14% answered “no” [other participants answered “don’t know”]). Responses to open-ended questions indicated that reductions in untargeted stimuli, especially in residents without dementia, led to understimulation, agitation, and increased mood problems. On the other hand, reductions in untargeted stimuli were reported to be beneficial for residents with advanced dementia, resulting in decreased restlessness. Participants stressed the importance of achieving an adequate balance between stimuli and tranquility, tailored to the individual, regardless of whether the individual had a dementia diagnosis.

Psychologist: People with advanced dementia benefited from the calmer living environment and the fact that this was more manageable.

Elderly care physician: For people without dementia, I expect more gloom and apathy; people with mild dementia also realize what is happening, so I expect the same from them. In advanced dementia, there is less awareness, and especially, due to controlled stimuli, less agitation.

Our analysis of the open-ended questions also suggests that apathetic behavior increased in all resident groups. In particular, participants reported that residents with agitated and psychotic behavior could benefit from strategies that limit untargeted stimuli, while residents with depressed or apathetic behavior may experience negative consequences from the use of such strategies.

#### Strategies

Participants indicated that they would like to continue using various strategies for reducing untargeted stimuli in the future (see Table 3). Specifically, 89% of participants responded positively to the idea of limiting the use of corridors adjacent to residents' rooms by suppliers and staff, while 78% responded positively to the idea of creating low-stimulation environments (i.e., environments with a low level of stimuli). Responses to open-ended questions indicate that participants especially endorse using these strategies with residents who have advanced dementia.

Activity therapist: In the case of dementia, we should offer major activities in a room located elsewhere in the nursing home. All the picking up and dropping off by employees and volunteers causes a lot of commotion.

**Table 3:** Opinions about whether to continue using various strategies for reducing untargeted stimuli in the future

	N	Yes	No	Don’t know
Creation of low-stimulation environments	189	148 (78.3%)	29 (15.3%)	12 (6.3%)
Limiting the use of corridors adjacent to residents' rooms by suppliers and staff	188	167 (88.8%)	13 (6.9%)	8 (4.3%)
Performing care actions in the room of the resident (instead of in a communal area)	188	121 (64.4%)	39 (20.7%)	28 (14.9%)
Not allowing visitors in communal areas anymore	189	105 (55.6%)	67 (35.4%)	17 (9.0%)
Setting visiting hours	189	59 (31.2%)	113 (59.8%)	17 (9.0%)
Regulation of times that suppliers are present in care units	185	142 (76.8%)	20 (10.8%)	23 (12.4%)

N participants (%). Participants were not obligated to answer these questions

Opinions were divided over whether visitors should be banned from the living room (56% answered “yes,” while 35% answered “no”) and whether visiting hours should be adopted (31% responded “yes,” while 60% responded “no”). Several participants believed these strategies may have added value for residents with advanced dementia but may result in a negative effect in residents without dementia. Too much walking in and out was regarded as disruptive, especially for residents with advanced dementia. Participants’ responses to open-ended questions suggested that, in general, fixed visiting hours are not desirable and that instead, there is more value in individualized arrangements or rules of conduct in the living rooms. For example, it was mentioned that such measures might stipulate the number of visitors, suitable visiting times, and the manner of interaction with other residents.

Psychologist: I am not in favor of set visiting hours, but I am in favor of establishing specific times when visits are not welcome, such as meal times, early mornings or later in the evenings.

Activity therapist: Setting visiting hours is not exactly person-oriented, but family members continually walking in and out can be very disruptive.

Most participants (64%) responded positively to the suggestion to perform care actions, such as setting up a wheelchair in the resident’s room and coordination between caregivers, in a private area, instead of in communal areas. Additional ideas for limiting untargeted stimuli in daily care included being alert to one's own and others' disturbing stimuli (e.g., wearing shoes with noisy soles or heels, talking loudly, walking unnecessarily) and limiting undesirable background noises (e.g., squeaky doors or cart wheels, loudly ringing telephones). On the other hand, participants stressed that some level of (untargeted) stimulation is desirable for many residents. It was noted that creating different sociotherapeutic environments indoors with different levels of stimulation (e.g., quiet/low stimulation, social/lively, or sensory stimulation) may be an appropriate way to meet the conflicting wishes and needs of individual residents. Organizing activities in separate rooms or creating rooms where residents can retreat with their loved ones may also help to create a person-oriented approach to satisfying the various differing needs of residents.

Psychologist: Oil squeaky cart doors and wheels, install sound-absorbing carpeting, and no dishwasher in the living room (a lot of noise from clattering plates and running the dishwasher).

Psychologist: Organize well-being activities at the care unit in such a way that residents are not always being picked up and dropped off from the living room. An activity itself, on the other hand, does provide stimuli in the living room, but these are desirable.

Elderly care physician: Furnishings (soothing colors, no busy patterns / frills). The basis of the environment should be low-stimulus, so that you can add stimuli depending on the group, as we already do: soft-soled shoes, no music while eating (or sometimes music, but only if the whole group finds having music more pleasant). Reasoning in terms music/activity should be based on the group that is there. Also important is for employees not to talk to residents about other residents, not to speak loudly, and not to chit-chat between themselves without also involving any residents in their vicinity. It’s their living room; you wouldn’t want this sort of thing happening in your home either.

In total, 154 out of 181 participants (85%) saw a role for themselves or their colleagues in continuing to limit untargeted stimuli. Respondents suggested the following as possible strategies for limiting untargeted stimuli: ensuring that members of the care team are aware of the need to limit untargeted stimuli; providing support for coaching staff and interventions; including details in the resident’s treatment plan that outline the types of untargeted stimuli to avoid; and, where possible, limiting one's own unintentional disruptive stimuli. Participants pointed to psychologists and occupational therapists as staff members who could play an important advisory or coaching role in reducing untargeted stimuli.

Psychologist: Make colleagues aware of how to limit these environmental stimuli. Consider providing a bit of coaching with regard to developing self-awareness.

### Targeted stimuli

A significant number of participants indicated that they would like to continue providing adjusted activities in the future (see Table 4). They were particularly positive about the use of small-scale activities (97% endorsed this strategy) and person-oriented activities (97% endorsed this strategy). Responses to open-ended questions emphasized the importance of encouraging social interaction and offering engaging stimuli, a strategy that can also involve simple, small-scale activities such as chatting or having a cup of coffee.

**Table 4:** Participant opinions about whether to continue specific adjustments to activities or new initiatives

	N	Yes	No	Don’t know
(More) small-scale activities / activities in smaller groups	175	170 (97.1%)	4 (2.3%)	1 (0.6%)
(More) activities in the living room	174	126 (72.4%)	32 (18.4%)	16 (9.2%)
(More) individual, person-oriented activities	174	168 (96.6%)	1 (0.6%)	5 (2.9%)
Attune to individual to provide more or fewer activities	175	158 (90.3%)	6 (3.4%)	11 (6.3%)
(Spontaneous) activities outside	174	153 (87.9%)	9 (5.2%)	12 (6.9%)
(Spontaneous) activities in the shared spaces	173	84 (48.6%)	64 (37.0%)	25 (14.5%)
Digital activities (e.g., online exercise program, virtual excursion, online bingo)	174	94 (54.0%)	56 (32.2%)	24 (13.8%)
Social robots or robot cuddly toys	175	111 (63.4%)	33 (18.9%)	31 (17.7%)

N participants (%). Participants were not obligated to answer these questions

Activity therapist: Activities offered in small groups have a calming effect and give clients confidence—they dare to engage more.

Activity therapist: It's about the little things—no need to celebrate with a carnival every day. It is about the sense of well-being that you get from the way the curtains are opened and the breakfast is presented. Getting back to basics is what’s important in life. Look more at individual needs.

Opinions were divided over the benefits of organizing (spontaneous) activities in the shared spaces (49% responded “yes,” while 37% responded “no”) and digital activities (54% answered “yes,” while 32% answered “no”). With regard to digital activities, participants responded to the open-ended questions that such activities seem less suitable for residents with dementia. However, cuddly robot toys were thought to induce a positive effect, especially in people with advanced dementia. Participants regarded both digital and in-person activities as valuable for residents without dementia.

Activity therapist: We can let residents do more with computers, etc. It is not the case that only people under the age of 75 enjoy playing interactive games or getting in touch with others. There is still much to gain here.

## Discussion

This survey study has shown that changes in stimuli brought about by the COVID-19 restrictions affected specific NH resident groups differently. While residents with advanced dementia and those with psychotic and agitated behavior seemed to benefit from the reduction of untargeted stimuli, residents without dementia and those with depressive and apathetic behavior may have been negatively affected when such stimuli were minimized. Various strategies to reduce untargeted stimuli that may be experienced as disruptive by residents or to adapt targeted stimuli seem to be beneficial, and this may continue to be the case in a post-pandemic era. On the other hand, some participants stressed that, for many residents, some amount of untargeted stimulation can be desirable.

These results confirmed and provided insight into previous findings with contradictory effects of the COVID-19 restrictions on challenging behavior in specific resident groups [5, 16, 17]. These contradictions might be explained by a heterogeneous NH population. The COVID-19 measures may have had beneficial effects for some groups and undesirable effects for others. The results are consistent with previous findings about untargeted stimuli being especially disturbing for residents with dementia and eliciting agitated behavior [2, 3, 23].

Our study suggests that consciously designing separate environments within the NH with different levels of stimulation (e.g., quiet/low stimulation, social/lively, or sensory stimulation) may be an appropriate way of meeting the opposing needs of residents [33–35]. Providing activities in small groups or in separate rooms may also be helpful in meeting the wishes and needs of individual residents. Although private bedrooms are generally available for most Dutch NH residents, providing separate living rooms where residents can retreat with their loved ones may also be desirable. In line with previous research [4, 36], our study highlights the value of meaningful and personally tailored activities to improving challenging behavior in NH care.

In general, our findings suggest the importance of paying attention to sensory information processing by individual residents and to different types of stimuli present within NHs. While sensory stimulation is important for NH residents [37–39], its effect on behavior may depend on the type of stimuli (targeted or untargeted at the resident) and characteristics of individual residents (e.g., ability to process sensory input). However, to date, little is known about the relation between sensory information processing and challenging behavior of NH residents. Based on our findings, particular attention should be paid to this in future research.

### Strengths and Limitations

The COVID-19 pandemic provided a unique situation for studying the effect of changes in diverse stimuli on challenging behaviors in NH residents. These changes would normally not have occurred. However, some of the changes in stimuli that we identified, such as limiting undesirable background noises or being alert to one’s own and others’ disturbing stimuli, might easily be integrated in care as usual as they are not directly related to the COVID-19 situation. Therefore, the findings can be generalized to a post-pandemic era to a certain extent. Furthermore, by including perspectives of participants from multiple disciplines—activity therapists, psychologists, elderly care physicians, and nurse specialists—we were able to achieve a broad understanding of the topic from different vantage points.

However, this study has several limitations as well. Due to nursing staff’s very high workload during the pandemic, we did not include them in the study. Residents themselves were also not included because of the ban on visitors in NHs. Although our aim was to learn from NH professionals’ experiences, input from the residents themselves, as well as those directly involved in their daily care, might have shed more specific light on the potential impacts of stimuli changes on residents.

Also, this study only analyzed perceptions of staff, without assessing actual effects of the changes in stimuli on challenging behavior. Although responses to both the closed and open-ended questions offered insight into the possible effects of these changes, as well as participants’ perceptions regarding the success of certain strategies, an objective measure for (changes in) challenging behavior in residents was not administered. It is also not clear to what extent changes in care actions by health care professionals in response to COVID-19 restrictions (e.g., offering more individual attention to residents to mitigate negative effects of the restrictions) or being more aware of residents’ behaviors, affected the perceptions of professionals. These actions may have also influenced residents’ challenging behavior, directly or indirectly.

Finally, we did not explicitly ask participants to verify the stage of dementia. We also did not ask participants to comment on possible effects of changes in stimuli on individual residents, but on the effect on groups of residents as a whole. These limitations also apply to the classification of challenging behavior. As our results may reflect opinions of professionals rather than objective assessments and are based on estimated effects on resident groups rather than individual residents, the results need to be interpreted with caution. Nonetheless, we believe the participants, who are all nursing home healthcare professionals, were able to provide their best informed opinion.

## Conclusions

This study provided insight into the observed effects of changes in stimuli on challenging behavior. Furthermore, potential strategies for managing challenging behavior in NHs were suggested in our study that may also be of use after the COVID-19 pandemic. The effects of COVID-19 restrictions in NHs highlight the importance of achieving a good balance between stimuli and tranquility, tailored to the level of stimulus processing and needs of individual residents. In addition, our study underlined challenges in balancing between individual needs and group home care. In general, stimuli in NHs seem to affect individual residents differently. Our findings suggest that the type of stimuli commonly present in NHs needs to be considered when analyzing and treating challenging behavior in practice. Different stimuli can have different effects on particular resident groups and specific types of challenging behaviors.

The study also showed that the pandemic may have served as a catalyst for new initiatives in NH care. To that end, new strategies and initiatives may prove to be beneficial after the pandemic. These findings can inform future interventions for successfully managing challenging behavior in NHs.

Nevertheless, more research is needed into the best way of meeting residents’ individual wishes and needs. In the post pandemic era, experimental studies that assess the effects of and experiences with suggested strategies and initiatives more thoroughly would be welcome. Furthermore, future research should include nursing staff as well as residents, so that different perspectives can be compared.

## Data Availability

A de-identified dataset used during the current study is available from the corresponding author upon reasonable request.

## Abbreviation

NH: Nursing Home
